# 3-Cyclo­hexyl­sulfinyl-5-iodo-2-methyl-1-benzofuran

**DOI:** 10.1107/S160053681101124X

**Published:** 2011-03-31

**Authors:** Hong Dae Choi, Pil Ja Seo, Byeng Wha Son, Uk Lee

**Affiliations:** a Department of Chemistry, Dongeui University, San 24 Kaya-dong Busanjin-gu, Busan 614-714, Republic of Korea; b Department of Chemistry, Pukyong National University, 599-1 Daeyeon 3-dong, Nam-gu, Busan 608-737, Republic of Korea

## Abstract

There are two independent mol­ecules, *A* and *B*, in the asymmetric unit of the title compound, C_15_H_17_ClO_2_S, in each of which the cyclo­hexyl ring adopts a chair conformation. The benzofuran units in each mol­ecule are essentially planar, with mean deviations from a least-squares plane defined by the nine constituent ring atoms of 0.006 (2) Å for *A* and 0.011 (2) Å for *B*. In the crystal, mol­ecules are linked by weak inter­molecular C—H⋯O and C—H⋯π inter­actions and by two I⋯O contacts [I⋯O = 3.079 (2) and 3.017 (2) Å].

## Related literature

For the pharmacological activity of benzofuran compounds, see: Aslam *et al.* (2009 ▶); Galal *et al.* (2009 ▶); Khan *et al.* (2005 ▶). For natural products with benzofuran rings, see: Akgul & Anil (2003 ▶); Soekamto *et al.* (2003 ▶). For structural studies of the related 5-bromo-3-cyclo­hexyl­sulfinyl-2-methyl-1-benzofuran, see: Choi *et al.* (2011 ▶). For a review of halogen bonding, see: Politzer *et al.* (2007 ▶).
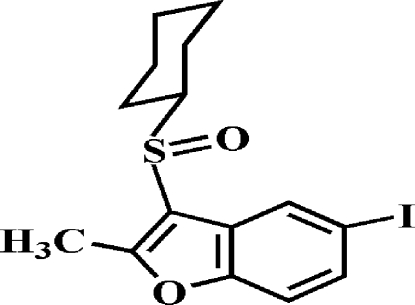


## Experimental

### 

#### Crystal data

C_15_H_17_IO_2_S
*M* *_r_* = 388.25
Monoclinic, 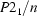

*a* = 14.1817 (2) Å
*b* = 12.1347 (2) Å
*c* = 18.1258 (3) Å
β = 101.136 (1)°
*V* = 3060.55 (8) Å^3^
*Z* = 8
Mo *K*α radiation
μ = 2.22 mm^−1^
*T* = 173 K
0.20 × 0.17 × 0.13 mm

#### Data collection

Bruker SMART APEXII CCD diffractometer
Absorption correction: multi-scan (*SADABS*; Bruker, 2009 ▶) *T*_min_ = 0.663, *T*_max_ = 0.758
30319 measured reflections
7599 independent reflections
6386 reflections with *I* > 2σ(*I*)
*R*_int_ = 0.034

#### Refinement

*R*[*F* ^2^ > 2σ(*F* ^2^)] = 0.031
*wR*(*F* ^2^) = 0.077
*S* = 1.04
7599 reflections
345 parameters
H-atom parameters constrained
Δρ_max_ = 2.47 e Å^−3^
Δρ_min_ = −1.61 e Å^−3^

### 

Data collection: *APEX2* (Bruker, 2009 ▶); cell refinement: *SAINT* (Bruker, 2009 ▶); data reduction: *SAINT*; program(s) used to solve structure: *SHELXS97* (Sheldrick, 2008 ▶); program(s) used to refine structure: *SHELXL97* (Sheldrick, 2008 ▶); molecular graphics: *ORTEP-3* (Farrugia, 1997 ▶) and *DIAMOND* (Brandenburg, 1998 ▶); software used to prepare material for publication: *SHELXL97*.

## Supplementary Material

Crystal structure: contains datablocks global, I. DOI: 10.1107/S160053681101124X/nk2093sup1.cif

Structure factors: contains datablocks I. DOI: 10.1107/S160053681101124X/nk2093Isup2.hkl

Additional supplementary materials:  crystallographic information; 3D view; checkCIF report

## Figures and Tables

**Table 1:** Hydrogen-bond geometry (Å, °) *Cg* is the centroid of the C16/C17/C22/O3/C23 furan ring.

*D*—H⋯*A*	*D*—H	H⋯*A*	*D*⋯*A*	*D*—H⋯*A*
C24—H24*A*⋯O4^i^	0.98	2.50	3.425 (4)	156
C29—H29*A*⋯*Cg*^i^	0.99	2.63	3.552 (4)	155

Symmetry code: (i) 
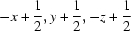
.

**Fig. 1.**
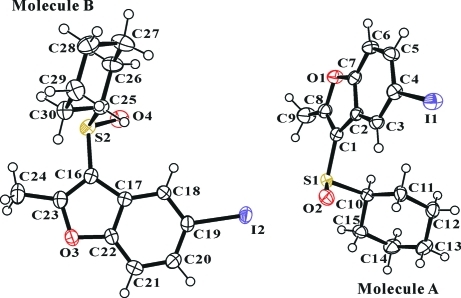
The molecular structure of the title compound. Displacement ellipsoids are drawn at the 50% probability level.

**Fig. 2.**
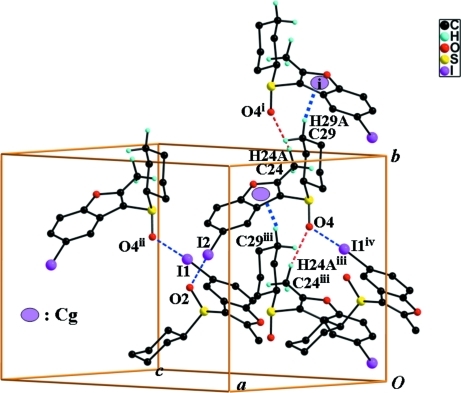
A view of the C—H···O, C—H···π and I···O interactions (dotted lines) in the crystal structure of the title compound. [Symmetry codes: (i) - x + 1/2, y + 1/2, - z + 1/2; (ii) x + 1, y, z; (iii) - x + 1/2, y - 1/2, - z + 1/2; (iv) x - 1, y, z.]

## supplementary crystallographic information

### Comment

Many compounds containing a benzofuran ring system exhibit interesting pharmacological properties such as antibacterial and antifungal, antitumor and antiviral, and antimicrobial activities (Aslam *et al.*, 2009, Galal *et al.*, 2009, Khan *et al.*, 2005). These compounds occur in a wide range of natural products (Akgul & Anil, 2003; Soekamto *et al.*, 2003). As a part of our ongoing study of the substituent effect on the solid state structures of 3-cyclohexylsulfinyl-5-halo-2-methyl-1-benzofuran analogues (Choi *et al.*, 2011), we report herein on the molecular and crystal structures of the title compound.

The asymmetric unit of the title compound is shown in Fig. 1. There are two independent unique molecules [labeled A & B] in which the benzofuran unit is essentially planar, with a mean deviation of 0.006 (2) Å for A and 0.011 (2) Å for B, respectively, from the least-squares plane defined by the nine constituent atoms. The cyclohexyl rings of both molecules adopt a chair conformation [endocyclic torsion angles are within a 51.5–59.0 (4)° range for A and 54.0–58.3 (4)° range for B, respectively].

In the crystal packing (Fig. 2), the B molecules are linked by weak intermolecular C—H···O hydrogen bonds between a methyl H atom and the O atom of the S═O unit (Table 1; C24—H24A···O4^i^), and by intermolecular C—H···π interactions between a cyclohexyl H atom and the furan ring (Table 1; C29—H29A···Cg^i^, Cg is the centroid of the C16/C17/C22/O3/C23 furan ring). Adjacent A and B molecules are linked by two I···O halogen bondings; the first one between the iodine and the O atom of the S═O unit [I1···O4^ii^ = 3.079 (2) Å; C4—I1···O4^ii^ =168.88 (9)°], and the second one between the iodine and the O atom of S═O unit [I2···O2 = 3.017 (2) Å; C19—I2···O2 = 175.89 (9)°] (Politzer *et al.*, 2007).

### Experimental

7% 3-chloroperoxybenzoic acid (224 mg, 1.0 mmol) was added in small portions to a stirred solution of 3-cyclohexylsulfanyl-5-iodo-2-methyl-1-benzofuran (335 mg, 0.9 mmol) in dichloromethane (40 mL) at 273 K. After being stirred at room temperature for 4h, the mixture was washed with saturated sodium bicarbonate solution and the organic layer was separated, dried over magnesium sulfate, filtered and concentrated at reduced pressure. The residue was purified by column chromatography (hexane-ethyl acetate, 2:1 v/v) to afford the title compound as a colorless solid [yield 76%, m.p. 412–413 K; *R*_f_ = 0.60 (hexane–ethyl acetate, 2:1 v/v)]. Single crystals suitable for X-ray diffraction were prepared by slow evaporation of a solution of the title compound in ethyl acetate at room temperature.

### Refinement

All H atoms were positioned geometrically and refined using a riding model, with C—H = 0.95 Å for aryl, 1.00 Å for methine, 0.99 Å for methylene and 0.98 Å for methyl H atoms, respectively. *U*_iso_(H) =1.2*U*_eq_(C) for aryl, methine and methylene, and 1.5*U*_eq_(C) for methyl H atoms.

### Crystal data

**Table:**

C_15_H_17_IO_2_S	*F*(000) = 1536
*M**_r_* = 388.25	*D*_x_ = 1.685 Mg m^−^^3^
Monoclinic, *P*2_1_/*n*	Mo *K*α radiation, λ = 0.71073 Å
Hall symbol: -P 2yn	Cell parameters from 9976 reflections
*a* = 14.1817 (2) Å	θ = 2.3–28.2°
*b* = 12.1347 (2) Å	µ = 2.22 mm^−^^1^
*c* = 18.1258 (3) Å	*T* = 173 K
β = 101.136 (1)°	Block, colourless
*V* = 3060.55 (8) Å^3^	0.20 × 0.17 × 0.13 mm
*Z* = 8	

### Data collection

**Table:**

Bruker SMART APEXII CCD diffractometer	7599 independent reflections
Radiation source: rotating anode	6386 reflections with *I* > 2σ(*I*)
graphite multilayer	*R*_int_ = 0.034
Detector resolution: 10.0 pixels mm^-1^	θ_max_ = 28.3°, θ_min_ = 1.7°
φ and ω scans	*h* = −18→18
Absorption correction: multi-scan (*SADABS*; Bruker, 2009)	*k* = −13→16
*T*_min_ = 0.663, *T*_max_ = 0.758	*l* = −24→23
30319 measured reflections	

### Refinement

**Table:**

Refinement on *F*^2^	Primary atom site location: structure-invariant direct methods
Least-squares matrix: full	Secondary atom site location: difference Fourier map
*R*[*F*^2^ > 2σ(*F*^2^)] = 0.031	Hydrogen site location: difference Fourier map
*wR*(*F*^2^) = 0.077	H-atom parameters constrained
*S* = 1.04	*w* = 1/[σ^2^(*F*_o_^2^) + (0.0307*P*)^2^ + 3.4955*P*] where *P* = (*F*_o_^2^ + 2*F*_c_^2^)/3
7599 reflections	(Δ/σ)_max_ = 0.001
345 parameters	Δρ_max_ = 2.47 e Å^−^^3^
0 restraints	Δρ_min_ = −1.61 e Å^−^^3^

### Special details

**Table:**

Geometry. All esds (except the esd in the dihedral angle between two l.s. planes) are estimated using the full covariance matrix. The cell esds are taken into account individually in the estimation of esds in distances, angles and torsion angles; correlations between esds in cell parameters are only used when they are defined by crystal symmetry. An approximate (isotropic) treatment of cell esds is used for estimating esds involving l.s. planes.
Refinement. Refinement of F^2^ against ALL reflections. The weighted R-factor wR and goodness of fit S are based on F^2^, conventional R-factors R are based on F, with F set to zero for negative F^2^. The threshold expression of F^2^ > 2sigma(F^2^) is used only for calculating R-factors(gt) etc. and is not relevant to the choice of reflections for refinement. R-factors based on F^2^ are statistically about twice as large as those based on F, and R- factors based on ALL data will be even larger.

### Fractional atomic coordinates and isotropic or equivalent isotropic displacement parameters (Å^2^)

**Table:**

	*x*	*y*	*z*	*U*_iso_*/*U*_eq_	
I1	1.048528 (14)	0.580334 (16)	0.151097 (11)	0.03378 (6)	
I2	0.606210 (13)	0.569038 (16)	0.364814 (11)	0.03435 (6)	
S1	0.72639 (5)	0.32745 (6)	0.29877 (4)	0.02762 (14)	
S2	0.22444 (5)	0.80407 (6)	0.18499 (4)	0.02861 (15)	
O1	0.68592 (14)	0.28895 (17)	0.07955 (10)	0.0312 (4)	
O2	0.77106 (16)	0.43298 (16)	0.33139 (12)	0.0354 (5)	
O3	0.26297 (14)	0.86421 (16)	0.40235 (11)	0.0322 (4)	
O4	0.24814 (16)	0.68779 (16)	0.16682 (12)	0.0369 (5)	
C1	0.73199 (18)	0.3275 (2)	0.20232 (15)	0.0263 (5)	
C2	0.79737 (18)	0.3824 (2)	0.16304 (14)	0.0237 (5)	
C3	0.87765 (19)	0.4506 (2)	0.18295 (15)	0.0260 (5)	
H3	0.9009	0.4713	0.2338	0.031*	
C4	0.9221 (2)	0.4868 (2)	0.12557 (16)	0.0286 (6)	
C5	0.8874 (2)	0.4594 (2)	0.05048 (15)	0.0298 (6)	
H5	0.9193	0.4868	0.0128	0.036*	
C6	0.8072 (2)	0.3928 (2)	0.02990 (15)	0.0294 (6)	
H6	0.7826	0.3741	−0.0211	0.035*	
C7	0.76537 (18)	0.3556 (2)	0.08750 (15)	0.0260 (5)	
C8	0.6672 (2)	0.2731 (2)	0.15035 (16)	0.0306 (6)	
C9	0.5859 (2)	0.1992 (3)	0.15600 (18)	0.0457 (8)	
H9A	0.6053	0.1224	0.1510	0.069*	
H9B	0.5311	0.2167	0.1158	0.069*	
H9C	0.5675	0.2094	0.2049	0.069*	
C10	0.81125 (18)	0.2169 (2)	0.33125 (14)	0.0255 (5)	
H10	0.7934	0.1514	0.2980	0.031*	
C11	0.91371 (19)	0.2497 (2)	0.32762 (17)	0.0304 (6)	
H11A	0.9293	0.3207	0.3540	0.037*	
H11B	0.9194	0.2599	0.2745	0.037*	
C12	0.9852 (2)	0.1619 (3)	0.36373 (17)	0.0357 (7)	
H12A	0.9738	0.0929	0.3343	0.043*	
H12B	1.0515	0.1870	0.3631	0.043*	
C13	0.9748 (2)	0.1400 (3)	0.44408 (17)	0.0392 (7)	
H13A	1.0215	0.0827	0.4664	0.047*	
H13B	0.9890	0.2081	0.4742	0.047*	
C14	0.8737 (2)	0.1017 (3)	0.44614 (18)	0.0404 (7)	
H14A	0.8677	0.0887	0.4990	0.048*	
H14B	0.8612	0.0311	0.4186	0.048*	
C15	0.7997 (2)	0.1864 (3)	0.41089 (15)	0.0325 (6)	
H15A	0.8066	0.2536	0.4425	0.039*	
H15B	0.7344	0.1564	0.4092	0.039*	
C16	0.26577 (18)	0.8201 (2)	0.28238 (15)	0.0253 (5)	
C17	0.34816 (19)	0.7692 (2)	0.32891 (15)	0.0246 (5)	
C18	0.42387 (18)	0.7019 (2)	0.31737 (15)	0.0254 (5)	
H18	0.4301	0.6803	0.2682	0.031*	
C19	0.48971 (19)	0.6675 (2)	0.38001 (16)	0.0284 (6)	
C20	0.4808 (2)	0.6979 (3)	0.45277 (17)	0.0369 (7)	
H20	0.5269	0.6728	0.4946	0.044*	
C21	0.4057 (2)	0.7641 (3)	0.46466 (17)	0.0362 (7)	
H21	0.3986	0.7846	0.5139	0.043*	
C22	0.34195 (19)	0.7987 (2)	0.40232 (16)	0.0283 (6)	
C23	0.2177 (2)	0.8744 (2)	0.32817 (16)	0.0296 (6)	
C24	0.1296 (2)	0.9429 (3)	0.3152 (2)	0.0410 (7)	
H24A	0.1470	1.0204	0.3248	0.061*	
H24B	0.0875	0.9192	0.3492	0.061*	
H24C	0.0958	0.9343	0.2630	0.061*	
C25	0.30894 (19)	0.8935 (2)	0.15006 (15)	0.0256 (5)	
H25	0.3751	0.8806	0.1798	0.031*	
C26	0.3067 (2)	0.8650 (3)	0.06778 (16)	0.0383 (7)	
H26A	0.3276	0.7877	0.0638	0.046*	
H26B	0.2403	0.8720	0.0389	0.046*	
C27	0.3730 (3)	0.9418 (3)	0.03484 (19)	0.0445 (8)	
H27A	0.4404	0.9276	0.0596	0.053*	
H27B	0.3670	0.9261	−0.0195	0.053*	
C28	0.3495 (3)	1.0609 (3)	0.04503 (18)	0.0402 (7)	
H28A	0.2855	1.0779	0.0143	0.048*	
H28B	0.3975	1.1080	0.0271	0.048*	
C29	0.3492 (2)	1.0873 (2)	0.12676 (18)	0.0355 (7)	
H29A	0.3300	1.1651	0.1310	0.043*	
H29B	0.4150	1.0782	0.1566	0.043*	
C30	0.2808 (2)	1.0129 (2)	0.15881 (17)	0.0305 (6)	
H30A	0.2841	1.0301	0.2126	0.037*	
H30B	0.2140	1.0254	0.1318	0.037*	

### Atomic displacement parameters (Å^2^)

**Table:**

	*U*^11^	*U*^22^	*U*^33^	*U*^12^	*U*^13^	*U*^23^
I1	0.03315 (11)	0.03675 (11)	0.03020 (10)	−0.01283 (8)	0.00306 (8)	−0.00015 (7)
I2	0.02812 (10)	0.03482 (11)	0.03866 (12)	0.00881 (7)	0.00288 (8)	−0.00231 (8)
S1	0.0232 (3)	0.0339 (3)	0.0270 (3)	0.0034 (3)	0.0080 (3)	−0.0011 (3)
S2	0.0251 (3)	0.0257 (3)	0.0328 (4)	−0.0052 (3)	0.0000 (3)	0.0027 (3)
O1	0.0288 (10)	0.0386 (11)	0.0257 (10)	−0.0108 (9)	0.0037 (8)	−0.0012 (8)
O2	0.0373 (12)	0.0329 (11)	0.0360 (11)	0.0062 (9)	0.0073 (9)	−0.0083 (9)
O3	0.0322 (10)	0.0312 (10)	0.0351 (11)	0.0073 (8)	0.0114 (9)	−0.0012 (9)
O4	0.0462 (12)	0.0228 (9)	0.0396 (12)	−0.0084 (9)	0.0032 (10)	−0.0031 (9)
C1	0.0226 (12)	0.0303 (13)	0.0254 (13)	0.0017 (11)	0.0033 (10)	0.0015 (11)
C2	0.0232 (12)	0.0237 (12)	0.0231 (12)	0.0029 (10)	0.0022 (10)	0.0005 (10)
C3	0.0281 (13)	0.0259 (13)	0.0233 (13)	0.0004 (11)	0.0031 (10)	−0.0013 (10)
C4	0.0275 (14)	0.0271 (13)	0.0310 (14)	−0.0040 (11)	0.0051 (11)	−0.0006 (11)
C5	0.0316 (14)	0.0345 (14)	0.0249 (13)	−0.0048 (12)	0.0092 (11)	0.0016 (11)
C6	0.0329 (15)	0.0324 (14)	0.0215 (13)	−0.0031 (12)	0.0016 (11)	−0.0016 (11)
C7	0.0221 (12)	0.0262 (13)	0.0278 (13)	−0.0030 (10)	0.0003 (10)	−0.0014 (11)
C8	0.0248 (13)	0.0368 (15)	0.0294 (14)	−0.0021 (12)	0.0034 (11)	0.0015 (12)
C9	0.0363 (17)	0.062 (2)	0.0374 (17)	−0.0221 (16)	0.0049 (14)	−0.0019 (16)
C10	0.0257 (13)	0.0273 (13)	0.0236 (12)	0.0010 (11)	0.0055 (10)	−0.0018 (10)
C11	0.0253 (13)	0.0341 (15)	0.0341 (15)	0.0047 (11)	0.0109 (11)	0.0094 (12)
C12	0.0300 (15)	0.0400 (16)	0.0381 (16)	0.0094 (13)	0.0085 (12)	0.0099 (13)
C13	0.0383 (17)	0.0414 (17)	0.0345 (16)	0.0015 (14)	−0.0019 (13)	0.0096 (14)
C14	0.0417 (18)	0.0476 (18)	0.0320 (16)	−0.0045 (15)	0.0071 (13)	0.0136 (14)
C15	0.0327 (15)	0.0411 (16)	0.0250 (13)	−0.0029 (13)	0.0089 (11)	0.0023 (12)
C16	0.0219 (12)	0.0233 (12)	0.0310 (14)	−0.0013 (10)	0.0058 (10)	0.0038 (11)
C17	0.0256 (13)	0.0216 (12)	0.0270 (13)	−0.0022 (10)	0.0059 (10)	0.0018 (10)
C18	0.0255 (13)	0.0260 (13)	0.0252 (13)	−0.0007 (10)	0.0060 (10)	−0.0016 (10)
C19	0.0243 (13)	0.0265 (13)	0.0341 (15)	0.0020 (11)	0.0049 (11)	−0.0024 (11)
C20	0.0354 (16)	0.0410 (17)	0.0314 (15)	0.0078 (13)	−0.0011 (12)	−0.0016 (13)
C21	0.0405 (17)	0.0422 (17)	0.0269 (14)	0.0037 (14)	0.0087 (12)	−0.0050 (12)
C22	0.0278 (13)	0.0266 (13)	0.0319 (14)	0.0030 (11)	0.0096 (11)	−0.0012 (11)
C23	0.0266 (13)	0.0255 (13)	0.0375 (15)	0.0005 (11)	0.0083 (12)	0.0022 (12)
C24	0.0331 (16)	0.0384 (17)	0.053 (2)	0.0119 (13)	0.0122 (15)	0.0045 (15)
C25	0.0258 (13)	0.0245 (12)	0.0256 (13)	−0.0034 (10)	0.0025 (10)	0.0017 (10)
C26	0.0544 (19)	0.0330 (15)	0.0278 (14)	−0.0071 (14)	0.0084 (13)	−0.0033 (12)
C27	0.064 (2)	0.0417 (18)	0.0322 (16)	−0.0072 (16)	0.0209 (16)	−0.0020 (14)
C28	0.0455 (19)	0.0386 (17)	0.0354 (16)	−0.0054 (14)	0.0051 (14)	0.0108 (13)
C29	0.0391 (17)	0.0233 (13)	0.0462 (18)	−0.0038 (12)	0.0133 (14)	−0.0001 (12)
C30	0.0299 (14)	0.0236 (13)	0.0382 (15)	−0.0021 (11)	0.0074 (12)	0.0001 (11)

### Geometric parameters (Å, °)

**Table:**

I1—C4	2.097 (3)	C13—H13A	0.9900
I1—O4^i^	3.079 (2)	C13—H13B	0.9900
I2—C19	2.100 (3)	C14—C15	1.520 (4)
I2—O2	3.017 (2)	C14—H14A	0.9900
S1—O2	1.499 (2)	C14—H14B	0.9900
S1—C1	1.765 (3)	C15—H15A	0.9900
S1—C10	1.822 (3)	C15—H15B	0.9900
S2—O4	1.502 (2)	C16—C23	1.344 (4)
S2—C16	1.760 (3)	C16—C17	1.441 (4)
S2—C25	1.819 (3)	C17—C18	1.397 (4)
O1—C7	1.372 (3)	C17—C22	1.397 (4)
O1—C8	1.373 (3)	C18—C19	1.387 (4)
O3—C22	1.373 (3)	C18—H18	0.9500
O3—C23	1.379 (3)	C19—C20	1.398 (4)
C1—C8	1.354 (4)	C20—C21	1.383 (4)
C1—C2	1.438 (4)	C20—H20	0.9500
C2—C7	1.395 (4)	C21—C22	1.370 (4)
C2—C3	1.397 (4)	C21—H21	0.9500
C3—C4	1.388 (4)	C23—C24	1.481 (4)
C3—H3	0.9500	C24—H24A	0.9800
C4—C5	1.395 (4)	C24—H24B	0.9800
C5—C6	1.386 (4)	C24—H24C	0.9800
C5—H5	0.9500	C25—C30	1.520 (4)
C6—C7	1.373 (4)	C25—C26	1.525 (4)
C6—H6	0.9500	C25—H25	1.0000
C8—C9	1.480 (4)	C26—C27	1.525 (4)
C9—H9A	0.9800	C26—H26A	0.9900
C9—H9B	0.9800	C26—H26B	0.9900
C9—H9C	0.9800	C27—C28	1.503 (5)
C10—C11	1.520 (4)	C27—H27A	0.9900
C10—C15	1.530 (4)	C27—H27B	0.9900
C10—H10	1.0000	C28—C29	1.516 (4)
C11—C12	1.528 (4)	C28—H28A	0.9900
C11—H11A	0.9900	C28—H28B	0.9900
C11—H11B	0.9900	C29—C30	1.520 (4)
C12—C13	1.515 (4)	C29—H29A	0.9900
C12—H12A	0.9900	C29—H29B	0.9900
C12—H12B	0.9900	C30—H30A	0.9900
C13—C14	1.515 (4)	C30—H30B	0.9900
			
C4—I1—O4^i^	168.88 (9)	C14—C15—H15A	109.3
C19—I2—O2	175.89 (9)	C10—C15—H15A	109.3
O2—S1—C1	107.12 (13)	C14—C15—H15B	109.3
O2—S1—C10	107.39 (12)	C10—C15—H15B	109.3
C1—S1—C10	99.60 (12)	H15A—C15—H15B	108.0
O4—S2—C16	106.17 (12)	C23—C16—C17	107.4 (2)
O4—S2—C25	107.09 (13)	C23—C16—S2	124.3 (2)
C16—S2—C25	99.73 (12)	C17—C16—S2	127.9 (2)
C7—O1—C8	106.7 (2)	C18—C17—C22	119.0 (2)
S1—O2—I2	105.19 (10)	C18—C17—C16	136.3 (2)
C22—O3—C23	106.1 (2)	C22—C17—C16	104.7 (2)
C8—C1—C2	107.3 (2)	C19—C18—C17	117.9 (2)
C8—C1—S1	122.3 (2)	C19—C18—H18	121.0
C2—C1—S1	130.3 (2)	C17—C18—H18	121.0
C7—C2—C3	119.1 (2)	C18—C19—C20	121.5 (3)
C7—C2—C1	105.0 (2)	C18—C19—I2	119.1 (2)
C3—C2—C1	135.9 (2)	C20—C19—I2	119.4 (2)
C4—C3—C2	117.3 (2)	C21—C20—C19	120.8 (3)
C4—C3—H3	121.3	C21—C20—H20	119.6
C2—C3—H3	121.3	C19—C20—H20	119.6
C3—C4—C5	122.0 (3)	C22—C21—C20	117.1 (3)
C3—C4—I1	119.9 (2)	C22—C21—H21	121.5
C5—C4—I1	118.0 (2)	C20—C21—H21	121.5
C6—C5—C4	121.2 (3)	C21—C22—O3	125.8 (3)
C6—C5—H5	119.4	C21—C22—C17	123.6 (3)
C4—C5—H5	119.4	O3—C22—C17	110.5 (2)
C7—C6—C5	116.1 (2)	C16—C23—O3	111.2 (2)
C7—C6—H6	122.0	C16—C23—C24	133.6 (3)
C5—C6—H6	122.0	O3—C23—C24	115.1 (3)
O1—C7—C6	125.5 (2)	C23—C24—H24A	109.5
O1—C7—C2	110.2 (2)	C23—C24—H24B	109.5
C6—C7—C2	124.2 (2)	H24A—C24—H24B	109.5
C1—C8—O1	110.8 (2)	C23—C24—H24C	109.5
C1—C8—C9	132.9 (3)	H24A—C24—H24C	109.5
O1—C8—C9	116.3 (2)	H24B—C24—H24C	109.5
C8—C9—H9A	109.5	C30—C25—C26	111.2 (2)
C8—C9—H9B	109.5	C30—C25—S2	109.18 (19)
H9A—C9—H9B	109.5	C26—C25—S2	108.34 (19)
C8—C9—H9C	109.5	C30—C25—H25	109.4
H9A—C9—H9C	109.5	C26—C25—H25	109.4
H9B—C9—H9C	109.5	S2—C25—H25	109.4
C11—C10—C15	112.5 (2)	C27—C26—C25	110.3 (2)
C11—C10—S1	111.76 (19)	C27—C26—H26A	109.6
C15—C10—S1	107.37 (18)	C25—C26—H26A	109.6
C11—C10—H10	108.4	C27—C26—H26B	109.6
C15—C10—H10	108.4	C25—C26—H26B	109.6
S1—C10—H10	108.4	H26A—C26—H26B	108.1
C10—C11—C12	111.1 (2)	C28—C27—C26	111.8 (3)
C10—C11—H11A	109.4	C28—C27—H27A	109.2
C12—C11—H11A	109.4	C26—C27—H27A	109.2
C10—C11—H11B	109.4	C28—C27—H27B	109.2
C12—C11—H11B	109.4	C26—C27—H27B	109.2
H11A—C11—H11B	108.0	H27A—C27—H27B	107.9
C13—C12—C11	110.8 (2)	C27—C28—C29	111.4 (3)
C13—C12—H12A	109.5	C27—C28—H28A	109.3
C11—C12—H12A	109.5	C29—C28—H28A	109.3
C13—C12—H12B	109.5	C27—C28—H28B	109.3
C11—C12—H12B	109.5	C29—C28—H28B	109.3
H12A—C12—H12B	108.1	H28A—C28—H28B	108.0
C14—C13—C12	110.2 (3)	C28—C29—C30	111.8 (3)
C14—C13—H13A	109.6	C28—C29—H29A	109.3
C12—C13—H13A	109.6	C30—C29—H29A	109.3
C14—C13—H13B	109.6	C28—C29—H29B	109.3
C12—C13—H13B	109.6	C30—C29—H29B	109.3
H13A—C13—H13B	108.1	H29A—C29—H29B	107.9
C13—C14—C15	111.3 (3)	C25—C30—C29	109.0 (2)
C13—C14—H14A	109.4	C25—C30—H30A	109.9
C15—C14—H14A	109.4	C29—C30—H30A	109.9
C13—C14—H14B	109.4	C25—C30—H30B	109.9
C15—C14—H14B	109.4	C29—C30—H30B	109.9
H14A—C14—H14B	108.0	H30A—C30—H30B	108.3
C14—C15—C10	111.7 (2)		
			
C1—S1—O2—I2	109.41 (11)	C11—C10—C15—C14	−51.5 (3)
C10—S1—O2—I2	−144.37 (10)	S1—C10—C15—C14	−174.9 (2)
O2—S1—C1—C8	−151.8 (2)	O4—S2—C16—C23	−139.2 (2)
C10—S1—C1—C8	96.5 (3)	C25—S2—C16—C23	109.7 (2)
O2—S1—C1—C2	25.9 (3)	O4—S2—C16—C17	32.9 (3)
C10—S1—C1—C2	−85.8 (3)	C25—S2—C16—C17	−78.2 (3)
C8—C1—C2—C7	0.0 (3)	C23—C16—C17—C18	178.7 (3)
S1—C1—C2—C7	−178.0 (2)	S2—C16—C17—C18	5.5 (5)
C8—C1—C2—C3	179.6 (3)	C23—C16—C17—C22	0.1 (3)
S1—C1—C2—C3	1.6 (5)	S2—C16—C17—C22	−173.1 (2)
C7—C2—C3—C4	−0.8 (4)	C22—C17—C18—C19	−0.1 (4)
C1—C2—C3—C4	179.6 (3)	C16—C17—C18—C19	−178.5 (3)
C2—C3—C4—C5	1.6 (4)	C17—C18—C19—C20	0.6 (4)
C2—C3—C4—I1	−175.79 (19)	C17—C18—C19—I2	−178.69 (19)
O4^i^—I1—C4—C3	134.7 (4)	C18—C19—C20—C21	−0.3 (5)
O4^i^—I1—C4—C5	−42.8 (6)	I2—C19—C20—C21	179.0 (2)
C3—C4—C5—C6	−1.0 (5)	C19—C20—C21—C22	−0.6 (5)
I1—C4—C5—C6	176.5 (2)	C20—C21—C22—O3	179.8 (3)
C4—C5—C6—C7	−0.5 (4)	C20—C21—C22—C17	1.2 (5)
C8—O1—C7—C6	−179.0 (3)	C23—O3—C22—C21	−177.6 (3)
C8—O1—C7—C2	−0.1 (3)	C23—O3—C22—C17	1.1 (3)
C5—C6—C7—O1	−179.9 (3)	C18—C17—C22—C21	−0.9 (4)
C5—C6—C7—C2	1.4 (4)	C16—C17—C22—C21	178.0 (3)
C3—C2—C7—O1	−179.6 (2)	C18—C17—C22—O3	−179.6 (2)
C1—C2—C7—O1	0.1 (3)	C16—C17—C22—O3	−0.8 (3)
C3—C2—C7—C6	−0.7 (4)	C17—C16—C23—O3	0.6 (3)
C1—C2—C7—C6	179.0 (3)	S2—C16—C23—O3	174.09 (19)
C2—C1—C8—O1	0.0 (3)	C17—C16—C23—C24	179.2 (3)
S1—C1—C8—O1	178.18 (19)	S2—C16—C23—C24	−7.3 (5)
C2—C1—C8—C9	177.0 (3)	C22—O3—C23—C16	−1.1 (3)
S1—C1—C8—C9	−4.8 (5)	C22—O3—C23—C24	−179.9 (2)
C7—O1—C8—C1	0.0 (3)	O4—S2—C25—C30	173.43 (18)
C7—O1—C8—C9	−177.5 (3)	C16—S2—C25—C30	−76.2 (2)
O2—S1—C10—C11	−42.6 (2)	O4—S2—C25—C26	52.1 (2)
C1—S1—C10—C11	68.8 (2)	C16—S2—C25—C26	162.5 (2)
O2—S1—C10—C15	81.1 (2)	C30—C25—C26—C27	57.2 (3)
C1—S1—C10—C15	−167.41 (19)	S2—C25—C26—C27	177.2 (2)
C15—C10—C11—C12	52.3 (3)	C25—C26—C27—C28	−54.6 (4)
S1—C10—C11—C12	173.2 (2)	C26—C27—C28—C29	54.0 (4)
C10—C11—C12—C13	−56.1 (3)	C27—C28—C29—C30	−55.8 (4)
C11—C12—C13—C14	59.0 (4)	C26—C25—C30—C29	−58.3 (3)
C12—C13—C14—C15	−58.2 (4)	S2—C25—C30—C29	−177.8 (2)
C13—C14—C15—C10	54.2 (4)	C28—C29—C30—C25	57.3 (3)

Symmetry codes: (i) *x*+1, *y*, *z*.

### Hydrogen-bond geometry (Å, °)

**Table:**

Cg is the centroid of the C16/C17/C22/O3/C23 furan ring.

**Table:**

*D*—H···*A*	*D*—H	H···*A*	*D*···*A*	*D*—H···*A*
C24—H24A···O4^ii^	0.98	2.50	3.425 (4)	156
C29—H29A···Cg^ii^	0.99	2.63	3.552 (4)	155

Symmetry codes: (ii) −*x*+1/2, *y*+1/2, −*z*+1/2.
